# Step-by-step iconographic description of a prolonged but still favourable course of orbital cellulitis in a child with acute rhinosinusitis: an iconographic case study

**DOI:** 10.1186/1824-7288-40-25

**Published:** 2014-03-04

**Authors:** Sara Torretta, Paola Marchisio, Michele Gaffuri, Pasquale Capaccio, Susanna Esposito, Lorenzo Pignataro

**Affiliations:** 1 Department of Clinical Sciences and Community Health, University of Milan, Fondazione IRCCS Ca’ Granda Ospedale Maggiore Policlinico, Via F. Sforza 35, 20122 Milano, Italy; 2 Department of Physiopathology and Transplantations, University of Milan, Fondazione IRCCS Ca’ Granda Ospedale Maggiore Policlinico, Milan, Italy; 3 Department of Surgical and Dental Biomedical Sciences, University of Milan, Fondazione IRCCS Ca’ Granda Ospedale Maggiore Policlinico, Milan, Italy

**Keywords:** Rhinosinusitis, Orbital cellulitis, Children

## Abstract

Orbital cellulitis is an infrequent complication of acute ethmoiditis possibly leading to life- or visual-threatening complications. Despite its natural history is well known, its clinical evolution may widely vary among patients, and even in the most favourable cases long-term sequelae may persist. We here provide a step-by-step iconographic description of a periorbital and orbital cellulitis occurring in a child with ipsilateral acute rhinosinusitis. Our report shows that an unusual long-term evolution of periorbital and orbital cellulitis is possible also in apparently favourable cases.

## Background

Orbital cellulitis is an infrequent complication of acute ethmoiditis that most frequently occurs in young children because of the thinner and dehiscent bone surface of their lamina papyracea and increased diploic venous supply in comparison with adulthood [1-4]. These conditions predispose children to infectious spreading from the ethmoid sinus to the near peri-orbital and orbital space, thus leading to a continuum of clinical disease patterns ranging from relatively mild periorbital cellulitis to a sight-threatening orbital abscess [1-4]. The latter may lead to optic neuropathy with visual loss, and predispose to life-threatening events such as cavernous sinus thrombosis, meningitis, and cerebral abscess [1-4].

Orbital cellulitis, which occurs in up to 35% of children with sinus-related orbital infections [5], is characterised by acute inflammation of intra-orbital fat without any evidence of abscess formation, and may lead to orbital displacement and proptosis, impaired extrinsic ocular motility with diplopia or, in the case of optic nerve involvement, reduced visual acuity [1,5,6]. The natural history of orbital cellulitis is well known [6-8], but its clinical evolution may vary widely from patient to patient and, even in the most favourable cases, long-term sequelae may persist and create disappointment and frustration for parents and clinicians.

We present a step-by-step iconographic description of the favourable but long-lasting course of a case of peri-orbital and orbital cellulitis in a child with acute ipsilateral rhinosinusitis.

## Case presentation

An 8-year-old Caucasion attended our Pediatric Emergency Department because of worsening left upper eyelid oedema and hyperaemia. Rhinitis and fever had begun two days before, and clarithromycin (15 mg/kg/day in two doses) had been prescribed by her primary care pediatrician and administered accordingly. The child was not affected by any systemic disease, was not allergic, and had never undergone nasosinusal surgery.

**Figure 1:**
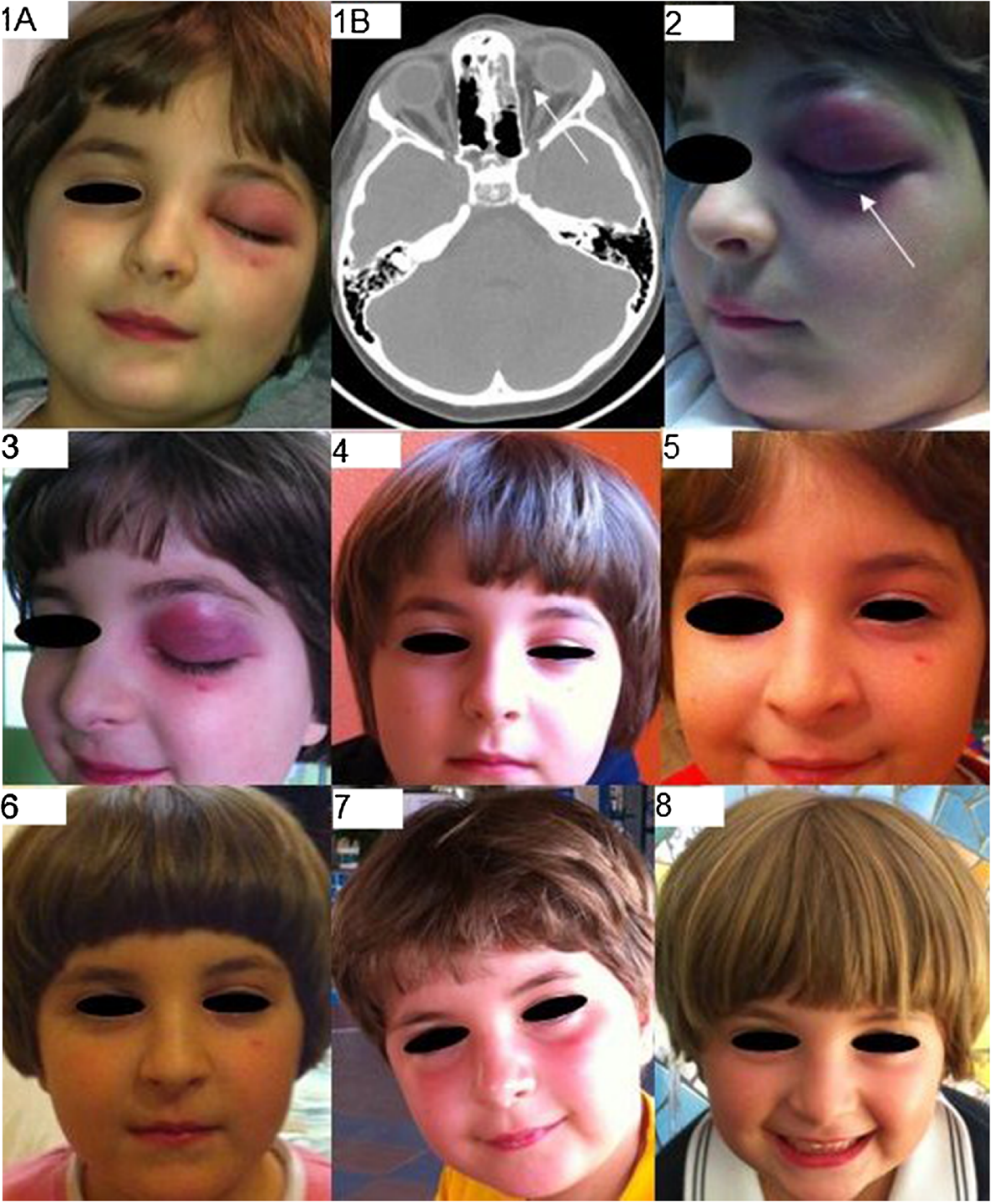
**Iconographic evolution of the case.** Panel **1**: At presentation. A: Moderate swelling and redness of the left upper eyelid, and slight involvement of the lower eyelid preventing orbital opening. B: Complete opacity of the left ethmoid with air-fluid levels, non-homogeneous intraorbital fat thickening mainly on the mesial side (arrow), and inflammatory involvement of the peri-orbital soft-tissues. Panel **2**: After two days. The upper eyelid redness and swelling slightly increased and there was a purulent conjunctival discharge (arrow). Panel **3**: After 10 days. Reduced eyelid swelling albeit with the persistence of redness. Proptosis solved. Panel **4**: After 40 days. Moderate upper eyelid swelling and redness persisted with asymmetrical in eyelid opening. Panel **5**: After 60 days. Persistence of moderate upper eyelid swelling but without Redness; asymmetrical eyelid opening. Panel **6**: After 90 days. Slight upper eyelid swelling with residual asymmetry in eyelid opening. Panel **7**: After 150 days. Minimal upper eyelid swelling with residual asymmetry in eyelid opening. Panel **8**: After 210 days. Complete clinical recovery.

Clinical assessment revealed moderate painful swelling and redness of the left upper eyelid that slightly involved the lower eyelid, caused proptosis and ophthalmoplegia, and prevented orbital opening (Figure 1, panel 1A).

The child was admitted to our Pediatric ward. Upon admission, flexible fiberendoscopy revealed oedema and hyperaemia of the nasal mucosa with congestion of the left infundibulum and a purulent discharge in the ipsilateral ostiomeatal complex and sphenoethmoidal recess. There were also non-obstructive adenoidal masses (grade II according to Cassano’s classification) [9] covered by mucous secretions.

The ophtalmologist documented moderate proptosis with a restricted upward gaze and conjunctival chemosis. No diplopia or reduced visual acuity were reported.

Maxillo-facial computed tomography showed a completely opaque left ethmoid, involvement of the maxillary and frontal sinusal sketches with air-fluid levels, and non-homogeneous intra-orbital fat thickening, mainly on the mesial side. No intra-orbital or sub-periostal abscesses, nor any intra-cranial complications were detected (Figure 1, panel 1B).

The only laboratory tests with pathological findings were and increased white blood cell count (13000/mm^3^) with neutrophilia (84%), and high levels of C-reactive protein (16 mg/dL). No immunodeficiency was detected.

On the basis of the diagnosis of orbital cellulitis complicating acute anterior left rhinosinusitis, the patient was hospitalised and intravenous treatment with piperacillin/tazobactam 150 mg/kg/day was begun and administered for ten days. After 48 hours, the upper eyelid redness and swelling had slightly increased. A purulent conjunctival discharge was noted but microbiological cultures did not reveal any pathogen (Figure 1, panel 2). During the following days, there was a progressive clinical improvement with the normalisation of laboratory test results, and the patients was discharged ten days later once the proptosis had disappeared (Figure 1, panel 3). At the time of discharge, oral cefuroxime axetil (30 mg/kg/day in two doses) was prescribed for a further seven days. The child underwent periodic clinical and nasal fiberendoscopic follow-up examinations (after 40, 60, 90 and 150 days) in the outpatient clinic of the Otolaryngology Department, and showed signs of a progressive improvement in upper eyelid swelling and redness, and eyelid opening (which the child’s parents reported to be particularly impaired in the evening) (Figure 1, panels 4–7) and the disappearance of orbital pain. The child was absent from school for 40 days. Nasal fiberendoscopy did not reveal any residual inflammation or infectious recurrences in the nasopharynx or nasosinusal district after 10 days.

A complete clinical recovery, with the normalisation of symmetrical eyelid opening was documented only seven months after the onset of the disease (Figure 1, panel 8). No recurrences were detected at the end of follow-up (25 months after disease onset).

## Conclusions

Sinus-related orbital involvement and its sequelae have been classified into five stages by Chandler *et al*. [10], with stage I corresponding to inflammatory oedema and stage V to cavernous sinus thrombophlebitis. In particular, the orbital complications of rhinosinusitis can be defined as peri-orbital cellulitis, orbital cellulitis, sub-periosteal abscess and orbital abscess depending on the site and entity of the inflammation. Peri-orbital cellulitis refers to the infection of tissues anterior to the orbital septum whereas, in the case of orbital cellulitis or abscess, the infection involves the posterior tissues; a sub-periosteal abscess is a pool of purulent material between the periorbita and the bony orbital wall under the periosteum [11]. Given the inflammatory involvement of the orbital contents posterior to the orbital septum, our patient belonged to stage II (orbital cellulitis) [11].

The pathogens mainly involved are those generally responsible for acute rhinosinusitis, such as *Streptococcus pneumoniae*, *Haemophilus influenzae*, *Moraxella catarrhalis*, *Staphylococcus aureus*, group A streptococcus, and upper respiratory tract anaerobes [12,13]. However, the introduction of the heptavalent pneumococcal vaccine and immunisation against *Haemophilus influenzae* type B have been associated with a decrease in the occurrence of invasive diseases such as peri-orbital and orbital cellulitis, and possibly a change in their microbiology [14,15].

Although uncommon (the reported incidence in specialised tertiary centres is 0.3-1.3 cases per month) [16], the disease processes may lead to serious complications, including vision loss and a predisposition for life-threatening events such as cavernous sinus thrombophlebitis or other intra-cranial complications [3,4,6,7]. The incidence of associated complications was especially high in the pre-antibiotic era, with 17% mortality due to meningitis and a 20% rate of blindness [8], the rates of vision loss and overall mortality are now respectively 11% and 2.5% [17]. In addition, there may be less troublesome but sometimes long-lasting functional and aesthetic sequelae, such as residual asymmetrical eyelid opening, impaired ocular motility, and eyelid inflammation.

In our case, although a clinical improvement was documented a few days after beginning adequate intravenous broad spectrum antibiotic therapy according to the guidelines [8], complete resolution of the clinical signs and symptoms of acute inflammation such as eyelid redness and swelling and orbital pain was only observed after 40 days, and complete clinical recovery with normally symmetrical eyelid opening was not documented until about seven months after disease onset. To the best of our knowledge, no similar cases have been reported in the literature.

There is no easy explanation for the prolonged course. In particular, conservative medical therapy (the first-line treatment for patients in Chandler stage II) [11] was administered in accordance with the guidelines [8,18], and no unfavourable clinical or anatomical situations coexisted. As a matter of fact, A CT scan failed to detect any condition that may have been responsible for impaired sinusal drainage or the persistence of orbital and peri-orbital inflammation, such as ethmoidal or orbital mucoceles or lamina papyracea erosion. Furthermore, no other risk factors were detected as the patient had never undergone nasosinusal surgery and immunodeficiency was excluded.

This long-term evolution resulted in discomfort for the patient and great concern for the parents and clinicians. However, the long-term serial clinical and instrumental follow-up with the collection and comparison of detailed iconography allowed us to assess the slow but progressive clinical improvement, and exclude the persistence or recurrence of orbital inflammation or the development of such subtle and often asymptomatic sequelae as mucoceles. The long-lasting clinical course initially led us to plan magnetic resonance imaging in order to rule out any orbital or ethmoidal mucocele, but the albeit slow progressive clinical improvement made this unnecessary. This approach seemed to be appropriate as no recurrences have been detected during the currently 25 months of follow-up.

Sinus-related orbital infections are well represented in the international scientific literature [1,2,5,8,11] but, to the best of our knowledge, the long-lasting course of pediatric peri-orbital and orbital cellulitis has not been previously assessed and there is no other detailed iconographic description of its temporal evolution.

In conclusion, we would like to emphasise the fact that the management of children with suspected sinus-related orbital infection is complex and requires a multidisciplinary approach involving pediatricians, otolaryngologists, ophthalmologists and radiologists. This not only allows a prompt diagnosis and adequate treatment, but also makes it possible to follow up and support children and their families during convalescence, and to detect any recurrence or related complications in a timely manner.

## Consent

Written informed consent was obtained from the patient’s parent for the publication of this report and any accompanying images.

## Competing interests

The authors declare that they have no competing interests.

## Authors’ contribution

ST and PM conceived the paper and drafted the manuscript. MG and PC performed acquisition of the data and helped to draft the manuscript. LP and SE participated in paper coordination and critically revised the manuscript for important intellectual contents. All the authors read and approved the final manuscript.
